# Potential Anti-Tumorigenic Properties of Diverse Medicinal Plants against the Majority of Common Types of Cancer

**DOI:** 10.3390/ph17050574

**Published:** 2024-04-30

**Authors:** Ghosoon Albahri, Adnan Badran, Zaher Abdel Baki, Mohamad Alame, Akram Hijazi, Anis Daou, Elias Baydoun

**Affiliations:** 1 Plateforme de Recherche et d’Analyse en Sciences de l’Environnement (EDST-PRASE), Beirut P.O. Box 657314, Lebanon; ghosoonalbahri@gmail.com (G.A.); alamefs@hotmail.com (M.A.); akram.hijazi@ul.edu.lb (A.H.); 2 Department of Nutrition, University of Petra Amman Jordan, Amman P.O. Box 961343, Jordan; abadran@uop.edu.jo; 3 College of Engineering and Technology, American University of the Middle East, Egaila 54200, Kuwait; zaher.abdelbaki@aum.edu.kw; 4 Pharmaceutical Sciences Department, College of Pharmacy, QU Health, Qatar University, Doha P.O. Box 2713, Qatar; 5 Department of Biology, American University of Beirut, Beirut 1107, Lebanon

**Keywords:** medicinal plants, availability, bioactive compounds, plant extraction, pharmacological activities, cancer treatment

## Abstract

Globally, cancer is one of the primary causes of both morbidity and mortality. To prevent cancer from getting worse, more targeted and efficient treatment plans must be developed immediately. Recent research has demonstrated the benefits of natural products for several illnesses, and these products have played a significant role in the development of novel treatments whose bioactive components serve as both chemotherapeutic and chemo-preventive agents. Phytochemicals are naturally occurring molecules obtained from plants that have potential applications in both cancer therapy and the development of new medications. These phytochemicals function by regulating the molecular pathways connected to the onset and progression of cancer. Among the specific methods are immune system control, inducing cell cycle arrest and apoptosis, preventing proliferation, raising antioxidant status, and inactivating carcinogens. A thorough literature review was conducted using Google Scholar, PubMed, Scopus, Google Patent, Patent Scope, and US Patent to obtain the data. To provide an overview of the anticancer effects of several medicinal plants, including *Annona muricata*, *Arctium lappa*, *Arum palaestinum*, *Cannabis sativa*, *Catharanthus roseus*, *Curcuma longa*, *Glycyrrhiza glabra*, *Hibiscus*, *Kalanchoe blossfeldiana*, *Moringa oleifera*, *Nerium oleander*, *Silybum marianum*, *Taraxacum officinale*, *Urtica dioica*, *Withania somnifera* L., their availability, classification, active components, pharmacological activities, signaling mechanisms, and potential side effects against the most common cancer types were explored.

## 1. Introduction

Cancer is the primary cause of death worldwide and a major barrier to extending human life expectancy [1]. According to updated estimates by the Global Cancer Observatory, in 2022, 10 cancer types accounted for approximately two-thirds of all new cases and deaths worldwide. Lung cancer accounted for 2.5 million new cases globally, or 12.4% of all new cases, making it the most common cancer. Next in order of incidence were colorectal cancer (1.9 million cases, 9.6%), prostate cancer (1.5 million cases, 7.3%), stomach cancer (970,000 cases, 4.9%), and female breast cancer (2.3 million cases, 11.6%). The most common cause of cancer-related deaths (1.8 million, or 18.7% of all cancer-related deaths) was lung cancer, which was followed by colorectal cancer (900,000, or 9.3%), liver cancer (760,000, or 7.8%), breast cancer (670,000, or 6.9%), and stomach cancer (660,000, or 6.8%) [2]. Globally, lung cancer is the leading cause of cancer-related mortality for men. In populations with lung cancer, the five-year survival rate ranges from 4 to 17%, depending on the stage and aggressiveness of the tumor. Significant advancements have recently been made in lung cancer screening, early diagnosis, and novel therapeutics [3]. Among adult females, breast cancer is currently the most common malignant tumor. It is the primary cause of cancer-related mortality for women worldwide. Drug therapy is still an effective way to treat breast cancer, even though early detection is still the best way to improve outcomes and survival. Antiestrogens are frequently used as the primary treatment because they are estrogen receptor (ER) positive in over 70% of cases of breast cancer. However, mounting data indicate that the most effective approach to managing breast cancer is to combine various medications [4]. Both men and women can develop colorectal cancer, which is one of the most common types of the disease. Patients rarely recover from it, and recurrence is frequent due to its malignant features [5]. The number of cancer cases is expected to rise rapidly in the ensuing decades as a result of changes in lifestyle and behavior, including obesity, smoking, physical inactivity, and reproductive patterns. Urbanization and economic growth are two other factors that contribute to different types of cancer. Tumors that do not respond to treatment are often caused by an alarming rate of drug resistance and extensive chemotherapy. Therefore, because of their low toxicity, high antitumor properties, and unique multi-targeting activities, natural products are becoming more and more popular as anticancer agents to treat drug-resistant malignancies [6,7,8]. The percentage of cancer cases worldwide that occur in low- and middle-income countries rose from 51% in 1975 to 55% in 2007 and is expected to reach 61% by 2050. It is no longer limited to lung, prostate, breast, tongue, ovarian, liver, skin, gastric, and colon cancer in Western industrialized nations; instead, it is now a global issue [9]. Various cancer treatments include immunotherapy, surgery, radiation therapy, and chemotherapy. Any of these are contingent upon the patient’s overall health status as well as the site, grade, and stage. In addition to eliminating cancerous cells, chemotherapy and radiation therapy also damage healthy cells. Solid tumors can be surgically removed from a patient’s body, but the quality of life is greatly diminished by numerous side effects. A range of medical combinations may be required to treat patients whose tumors become resistant to chemotherapeutic treatments. Many technologies are presently being tested in clinical trials, particularly nanomedicines, which are also advancing the creation of biocompatible materials for use in cancer treatment and diagnosis, like the creation of novel medications that treat different forms of cancer by utilizing ethnomedicinal plants [10,11]. The use of natural, synthetic, or biological agents that can reverse, suppress, or prevent carcinogenesis or the progression of premalignant cells toward invasive tumors has made cancer chemoprevention an important therapeutic option that may help fight cancer [12,13]. Plants and products derived from them have promising potential as a source of less toxic anticancer drugs because they contain a variety of phytochemicals that have been isolated from various parts and have been demonstrated to have anticancer properties. It has been demonstrated that these substances stop the growth of cancer cells, trigger apoptosis, and disrupt the cell cycle [14,15]. This review summarizes the anticancer therapeutic potential as well as the side effects of these medicinal plants, their availability, extraction methods, active constituents, anticancer pharmacological efficiency, and patents related to their anticancer effects. The goal is to attract attention to the beneficial pharmacological properties of some of the most effective medicinal plants, including *Annona muricata*, *Arctium lappa*, *Arum palaestinum*, *Cannabis sativa*, *Catharanthus roseus*, *Curcuma longa*, *Glycyrrhiza glabra*, *Hibiscus*, *Kalanchoe blossfeldiana*, *Moringa oleifera*, *Nerium oleander*, *Silybum marianum*, *Taraxacum officinale*, *Urtica dioica*, and *Withania somnifera* L., and encourage scientists and chemists to formulate more effective medications using these medicinal herbs. The effectiveness of these medicinal plants against various cancer types is displayed in Table 1, and Table 2 lists the main active ingredients found in various plant parts that are responsible for this efficacy.

## 2. Methods

The current review focuses primarily on the original, peer-reviewed, and generally unbiased publications that have been published, as well as patents, linking various medicinal plants to their pharmaceutical qualities to different types of cancers between 2000 and 2024 but mainly focusing on the most recent papers. Related keywords such as “medicinal plants”, “distribution”, “bioactive components”, “extraction methods”, “pharmacokinetics”, “signaling pathways”, “pharmacological mechanism of action”, “cancer”, “chemoprevention”, “in vitro”, “in vivo”, “clinical trials”, and “side effects” were searched to find data (Google Scholar, PubMed, and Scopus) and patents (US patent, Patent scope, and Google Patent).

### 2.1. Annona muricata

**Figure 1:**
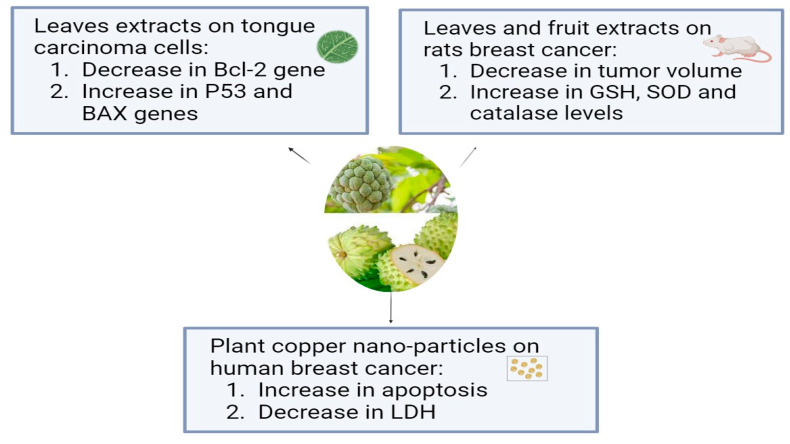
The antiproliferative effects of *Annona muricata* L. on different types of cancer cell lines.

*Annona muricata*, which is known as Soursop or Graviola, is a tropical plant belonging to the Annonaceae family. For thousands of years, *A. muricata* has been a staple of traditional medicine due to its well-known medicinal qualities, and it is extensively grown all over the world in tropical and subtropical climates. The aerial parts of graviola serve multiple purposes: the fruits are widely used as confections, and many preparations, particularly decoctions of the bark, fruits, leaves, pericarp, seeds, and roots, are widely used in traditional medicine by local communities in tropical Africa and South America to treat a variety of illnesses, including cancer [69]. In addition to carbohydrates and bioactive substances, like acetogenins, alkaloids, and phenolic compounds, like gallic acid and quercetin, which have strong biological and antioxidant properties, it has more than 20–35 mg of vitamin C per 100 g of soursop [16]. Using in vitro tests, the aqueous leaf extract of *Annona muricata* (ALEAM) showed cytotoxic, antiproliferative, anti-metastatic, and pro-apoptotic effects on the oral tongue squamous cell carcinoma cell line SCC-15. The findings demonstrated that ALEAM inhibited colony formation and cell migration, in addition to displaying significant cytotoxic activity in a dose-dependent manner. A highly significant decrease in Bcl-2 gene expression and a highly significant increase in P53 and Bax gene expression in the study group relative to the control group confirmed the pro-apoptotic characteristics [70]. A previous study compared the effects of *A. muricata* fruit and leaf extracts on rats’ breast cancer induced by DMBA. The aqueous extract of *A. muricata* fruit at 200 mg/kg (3 days/week or daily) and the ethanolic extract of *A. muricata* Linn leaves at 200 mg/kg daily were administered to rats exposed to DMBA (50 mg/kg). The normal and diseased controls received a vehicle, while the positive control group was given tamoxifen at a dose of 3.3 mg/kg. When compared to the DMBA group, *A. muricata* extracts (leaves and fruit) and tamoxifen significantly decreased tumor volume and weight, death and tumor incidences, total protein, and CA15-3 levels. When compared to DMBA, they showed antioxidant activity as evidenced by elevated GSH, SOD, and catalase levels and decreased MDA levels, where the fruit extract in water had a stronger anti-breast cancer effect than the leaf extract in ethanol [71]. Another study has examined, for the first time, a straightforward biosynthesis method for producing copper oxide nanoparticles (CuO NPs) and testing their anticancer properties using an extract from the plant *Annona muricata* L. The fruits’ seedless endocarp was shade-dried after the outer epicarp was removed, and 50 g of the dried endocarp were macerated into a powder. To make a 5% (*w*/*v*) suspension, the fruit powder was mixed with hot boiling distilled water and shaken at 200 rpm for four hours at 37 °C. After allowing the suspension to reach room temperature, four layers of No. 1 Whatman filter paper and a 0.22 m filter (Millipore, Sigma, St. Louis, MO, USA) were added. The aqueous extract was filtered and freeze-dried, and the powder was stored at −20 °C until needed. The human breast epithelial cell line (HBL-100) and the breast cancer cell line (AMJ-13) were used to test the synthesized nanoparticles’ antiproliferative qualities. CuONPs inhibited AMJ-13 and MCF-7 cell proliferation, according to this study. The results imply that the generated copper oxide nanoparticles inhibited the proliferation of particular cell lines found in breast cancer. It showed that CuONPs were absorbed by cancer cells and caused apoptosis. Additionally, treatment with CuONPs increased the production of lactate dehydrogenase (LDH), most likely as a result of damage to the cell membrane that resulted in leaks containing substances found in cells, such as lactate dehydrogenase [72]. According to a study in which semi-purified fractions of the ethyl acetate extract from *Annona muricata* L. (guyabano) leaves were tested for cytotoxicity against A549 lung cancer cells after being purified using column chromatography, guyabano leaves have anticancer properties against lung cancer cells. In the MTS test, fractions F15–16C and F15–16D showed the strongest anticancer activity, with cytotoxicity percentages of 99.6% and 99.4%, respectively [73]. The anticancer effects of *Annona muricata* L. are shown in Figure 1.

### 2.2. Arctium lappa L.

*Arctium lappa* L., also known as burdock, is a traditional Chinese plant that belongs to the Asteraceae family and is grown extensively in the Chinese regions of Xuzhou and Linyi. It has several useful ingredients, including inulin, phenolic acids, and dietary fibers. About 10% of the weight of burdock root is soluble dietary fiber and it is a good source of pectic polysaccharides [74]. Numerous bioactive components found in burdock, such as polysaccharides, polyphenols, flavonoids, and volatile oils, support the plant’s biological properties, which include anti-inflammatory, antioxidant, antibacterial, and antiviral effects [75]. Previous research has shown that *Arctium lappa* is the natural source of a lignan called lappaol F (LAF). For 48 h, three different colon cancer cell lines—SW480, HCT15, and HCT116—were exposed to LAF at concentrations of 25, 50, and 75 μmol/L. LAF caused cell cycle arrest at the S phase and inhibited the growth of several colon cancer cell lines, including SW480, HCT15, and HCT116 cells (IC50 47.1, 51.4, and 32.8 μmol/L, respectively). Also, The S-phase cell cycle arrest and proliferation inhibition brought on by LAF was lessened by CDKN1C/p57 knockdown [76]. *Arctium lappa* is the source of arctiin, a lignan glycoside. A recent study showed that in human cervical cancer cells, actin significantly inhibited cell migration and invasion while exhibiting low cytotoxicity. After arctiin treatment, the S100A4 protein expression and mRNA levels in HeLa and SiHa cells were significantly decreased. Additionally, by suppressing S100A4 and the PI3K/Akt pathway, arctiin prevents the migration and invasion of cervical cancer cells [77]. The aerial parts of *Arctium lappa* L. were fractionated using a bioactivity-guided method in a previous study. The extracts were then tested in vitro on normal cells (EA.hy926), colorectal cancer (HCT-116), and breast cancer (MCF-7). Strong activity against the MCF-7 and EA.hy926 cell lines was demonstrated by the n-hexane fraction (EHX) of the ethanolic extract (IC50 values: 14.08 ± 3.64 and 27.25 ± 3.45 μg/mL, respectively). When the signal transduction pathways of cancer cells were examined, EHX considerably increased the expression of p53, TGF-β, and NF-κB. Furthermore, it was discovered that EHX inhibits cell proliferation, migration, invasion, and colonization, thereby upsetting the breast cancer cells’ metastatic cascade [78].

### 2.3. Arum palaestinum

**Figure 2:**
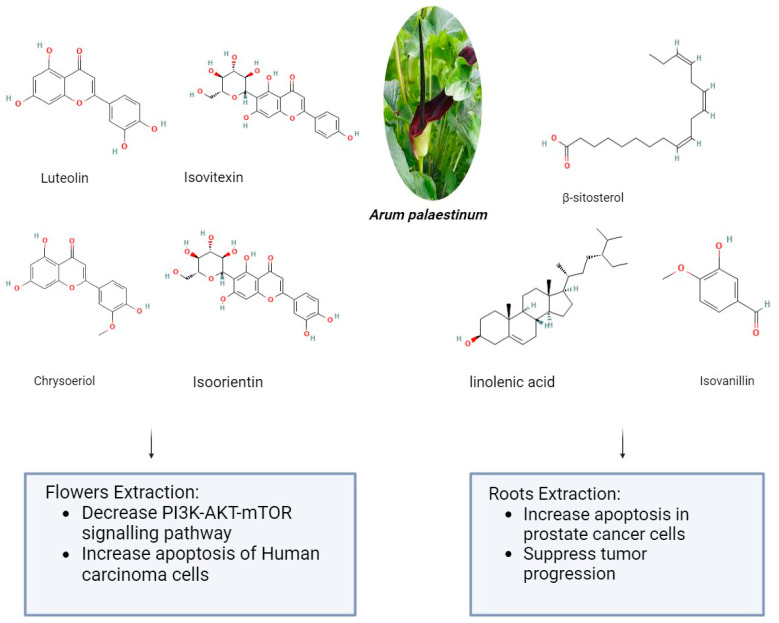
The anticancer effect of *Arum palaestinum* flower and root extractions (The molecular structures of the chemical compounds were retrieved from https://pubchem.ncbi.nlm.nih.gov/, accessed on 21 April 2024).

*Arum palaestinum* is one of the 26 species of the *Arum* genus, which is a member of the Araceae family. The genus *Arum* L. comprises 26 species of flowering plants that are found in Northern Africa, the Mediterranean region, Western Asia, and Europe. They are members of the Araceae family of flowering plants. The presence of alkaloids, proanthocyanidins, flavones, and their C-glycosides and flavonols are its defining characteristics. It may be found as an ornamental plant in gardens in non-endemic areas [79]. *Arum palaestinum* Boiss (*A. palaestinum*), commonly referred to as the black calla lily, is edible, regarded as an ornamental plant, and has been used in traditional medicine to treat several chronic illnesses, including diabetes, atherosclerosis, cancer, and stomach acidity [80]. The aerial parts of *A. palaestinum* were air-dried and ground in previous research. Then, three extractions at room temperature with 70% methanol were made three days apart from each other from the resulting powder (1450 g). To obtain a residue of 302 g, the aqueous methanol extract was evaporated at a lower temperature and pressure. Dissolving the residue in 500 mL of distilled water allowed for fractionation using diethyl ether (44 g), dichloromethane (26 g), ethyl acetate (40 g), butanol (75 g), methanol (56 g), and water (58 g) by their polarity. After that, For the fractionated extract and isolated compounds, in vitro cytotoxic activity was examined against four human carcinoma cell lines: Hep2, HeLa, HepG2, and MCF7. However, because the diethyl ether fraction had the strongest cytotoxic effect, it was subjected to GC-MS analysis to determine which active ingredients were responsible for the cytotoxic activities. From the diethyl ether and ethyl acetate, four flavonoid compounds—luteolin, chrysoeriol, isoorientin, and isovitexin—were separated. Against every cell line under investigation, the extracts and the pure isolated compounds demonstrated markedly strong antiproliferative activity [81]. The impact of *Arum palaestenium* (AP) on hepatocellular carcinoma was evaluated through analyses of its effects on the PI3K-AKT-mTOR molecular signaling pathway, cell cycle, proliferation (CFSE), apoptosis (annexin-v+/PI), and tumorigenicity (αFP and HbsAg). After the dried AP flowers were finely chopped, 100 g were boiled in 1 L of distilled water until only ¼ of their original volume remained. The decoction was then filtered, and the resulting filtrate was then put in a freeze-dryer device to solidify the aqueous extract into powder. A recent study showed the impact of *Arum palaestenium* (AP) on hepatocellular carcinoma through analyses of its effects on the PI3K-AKT-mTOR molecular signaling pathway, cell cycle, proliferation (CFSE), apoptosis (annexin-v+/PI), and tumorigenicity (αFP and HbsAg). After the dried AP flowers were finely chopped, 100 g were boiled in 1 L of distilled water until only ¼ of their original volume remained. The decoction was then filtered, and the resulting filtrate was then put in a freeze-dryer device to solidify the aqueous extract into powder. The anticancer results showed that incubation with AP fractions delayed the cell cycle as evidenced by decreased cell proliferation rates; the effect of the aqueous fraction was most noticeable in a delay in the S phase. The flower extract in methanol accelerated the cells in the G2-M phase, indicating that AF flower extracts may have anticancer properties. The aqueous extract of AP (1) decreased secretions of HCC αFP by 1.55-fold and 3.3-fold at the 50 and 100 μg/mL concentrations, respectively (*p* = 0.0008); (2) decreased phosphorylation in the PI3K-AKT-mTOR signaling pathway (*p* < 0.05); and (3) shifted cells from necrosis to apoptosis [82]. About 14 g of *Arum palaestinum* Boiss leaves and roots were boiled for 15 min in 1 L of water, then the heat was turned down for an additional 15 min. The plant extract used in the testing was obtained by filtering the mixture to remove big particles. It is challenging to regulate precisely how much of each chemical component there is in plant extracts because plants vary greatly in this regard. The extract’s chemical components have been discussed elsewhere. After three of the chemical components were strengthened to known levels, a compound known as “GZ17” was produced. 25 g of isovanillin, linolenic acid, and β-sitosterol (Sigma-Aldrich, St. Louis, MO, USA) were combined with 3.79 L of water to create the *Arum palaestinum*. This resulted in a final concentration of 43.3 μM isovanillin, 23.8 μM linolenic acid, and 15.9 μM β-sitosterol. After being diluted in purified water and sonicated to produce a 50 mg/mL stock solution, GZ17 was diluted in water to the appropriate concentrations. To find out the impact of an extract made from boiling *Arum Palaestinum* Boiss roots, verified prostate cancer cells were plated as three-dimensional spheroids. Furthermore, male 8-week-old NU/NU mice bearing xenograft tumors originating from the prostate cancer cell line were administered 1000 mg/kg body weight gavage of the GZ17 suspension daily. At the end of the three-week study, the tumor growth was measured several times using calipers, and the removed tumors were weighed. The vehicle was given to the control mice (10 mice per group) in the same way and volume. The extract (known as GZ17) was fortified with more isovanillin, linolenic acid, and β-sitosterol, which had a greater effect on the rate of cell death. At the highest dose, the percentage of dead cells increased from 30% to 55%, while the vehicle control did not affect the number of cells. At doses toxic to treated cancer cells, GZ17 did not cause any cell death when applied to non-cancerous tissue, in this case, human islets. Tumor growth in the control group increased gradually over the course of three weeks, but tumors in NU/NU mice with xenograft prostate tumors treated with GZ17 showed a striking suppression of tumor progression as shown in Figure 2 [83]. However, some minor clinical manifestations were reported after plant administration, mainly erythema and mouth irritation, agitation, and drooling [79].

### 2.4. Cannabis sativa

*Cannabis sativa* is an annual herb that is also known as Indian hemp or *Cannabis indica*. It is in the Cannabaceae family. Since 900 BC, people have been cultivating the plant, which is native to Central Asia, primarily for medical purposes [84]. *Cannabis* contains compounds called cannabinoids (CBs), including terpenes, cannabidiol (CBD), and Δ9-tetrahydrocannabinol (Δ9-THC). Cannabis is used to treat cancer pain, cachexia, sleeplessness, and anxiety and suppress tumors in cancer patients [85]. Endogenous ligands, metabolizing enzymes, and cannabinoid receptors (CB1, CB2) make up the endocannabinoid system (ECS), which is how cannabinoids work. Several countries have recently legalized the use of *Cannabis* for medical purposes. Numerous preclinical investigations have shown that cannabinoids may be able to treat cancers of the breast, colorectum, pancreas, cervix, prostate, lymphoma, and leukemia [86]. Additionally, it has been demonstrated that elevated levels of circulating endocannabinoids are linked to accelerated disease progression in both humans and the mouse model of metastatic melanoma. Additionally, it has been reported that cannabinoids prevent PC-3 cell proliferation induced by nerve growth factor (NGF). This is achieved through their interaction with CB1 receptors and their stimulation of TRPV1 channels in synthetic endocannabinoid-vanilloid hybrids. Other research, however, showed that the relationship between TRPV1 receptors and cannabinoid-induced antitumor activity was only tangentially related [87]. The body produces lipid-based neurotransmitters called endocannabinoids, such as anandamide and 2-arachidonoylglycerol (2-AG), which bind to the CB1 and CB2 cannabinoid receptors on cell surfaces to cause a response from the cell. After binding to CB1 and CB2 receptors and emulating endocannabinoids, cannabinoids can then act on the ECS. G-protein-coupled receptor 55 (GPR55), transient receptor potential cation channel subfamily V member 1 (TRPV1), TRPV2, transient receptor potential cation channel subfamily M member 8 (TRPM8), and peroxisome proliferator-activated receptors (PPARs) are among the other receptors that interact with endocannabinoids and modulate the ECS. It is noteworthy that colorectal cancer (CRC) cells and tissues exhibit the expression of CB1 and CB2 receptors, as well as TRPM8, TRPA1, TRPV1, and TRPV2 receptors. These receptors can be bound by cannabinoids to cause biological effects on colorectal cancer [88,89]. A prior investigation examined the effects of cannabbidiol CBD on the novel pro-apoptotic Noxa-reactive oxygen species (ROS) signaling pathway in colorectal cancer (CRC) cells. In CBD experiments, the CRC cell lines DLD-1 and HCT116 were used. By controlling a wide range of pro- and anti-apoptotic proteins, of which Noxa exhibited noticeably greater expression, CBD caused apoptosis. Consequently, CBD induced apoptosis in a way that was dependent on ROS and Noxa. Together, the study’s findings validated CBD’s status as a cutting-edge, trustworthy anticancer medication by reiterating the effects of CBD treatment in vivo [90]. Other research investigated the possibility of extracting bioactive compounds from industrial light *Cannabis* production waste without the need for additional chemicals. Leaves, flowers, and fibers from *Cannabis sativa* L. waste were used. The wastes were crushed into small pieces with gentle pressure to make the extraction process easier. After a short 72-h maceration period in which water was the only solvent, two extracts were obtained by mechanical extraction. Before the extraction, the waste material was pre-treated using a microwave energy (MWE)-assisted procedure. The other two extracts were obtained by mechanical extraction without any pre-treatment following one and two months of water maceration, respectively. In addition to the mechanical extraction process, brief maceration and protein precipitation were used to produce the final extract. The antimicrobial activity of the five obtained extracts was evaluated on HT-29 colon cancer cells under oxidative stress, as well as on planktonic and sessile cells of pathogenic strains of *Candida albicans*, *Candida parapsilosis*, and *Candida tropicalis*. The findings showed that these extracts have intriguing qualities that prevent the growth of fungal biofilms and function as antioxidants, opening the door for more research into the environmentally friendly valorization of hemp waste for various biomedical uses [91]. However, it is crucial to remember that *Cannabis* use can also have negative effects on health, including fatigue, tachycardia, nausea, dizziness, dry mouth, altered mood and behavior, psychomotor impairment, and auditory and visual hallucinations. In addition, prolonged use of medicinal cannabis may raise the chance of developing psychiatric comorbidities and substance use disorders. It is also critical to keep in mind that marijuana use is still illegal in several nations where it is still regulated [92,93].

### 2.5. Catharanthus roseus

**Figure 3:**
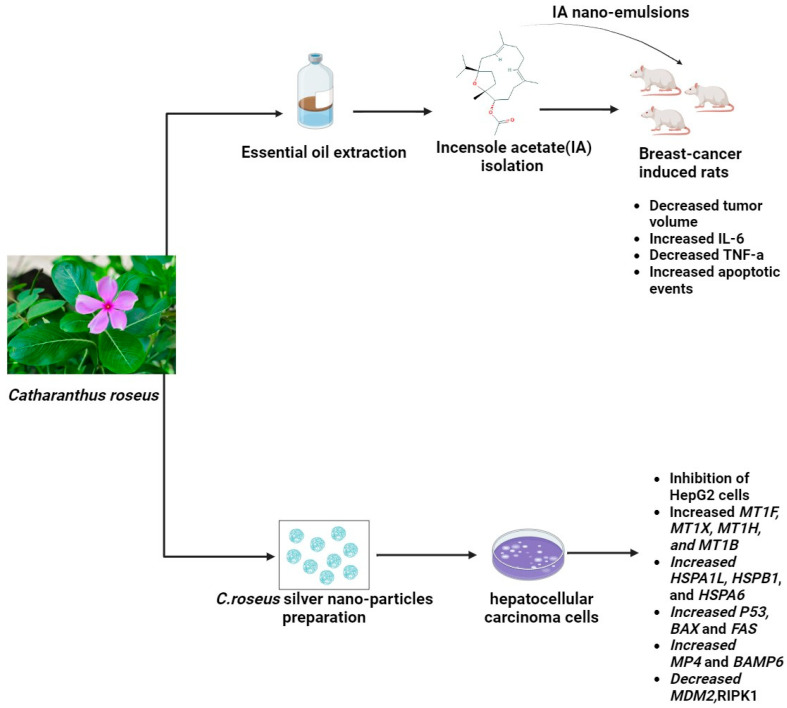
The potential anticancerogenic effect of *Catharanthus roseus* nano-emulsions and nano-particles (The molecular structure of the chemical compounds were retrieved from https://pubchem.ncbi.nlm.nih.gov/, accessed on 21 April 2024).

*Catharanthus roseus* (L.) is alternatively known as Madagascar periwinkle. Under the genus *Catharanthus* in the family Apocynaceae, this plant is classified as *C*. *roseus*. Steroids, flavonoids, and alkaloids have been identified as the active chemical constituents of this plant. Geographically, it is believed that *C. roseus* Linn. inhabits Madagascar, an island in the Indian Ocean. Most tropical and subtropical regions of the world now have a common distribution of the plant. Ajmalicine (raubasine), serpentine, and reserpine are the principal alkaloids found in the roots of *C. roseus* Linn. Its basal stems have been found to contain ajmalicine, Vincente, vincamine, raubasine, reserpine, catharanthine, and other compounds. Its flowers primarily contain coronaridine, tetrahydroalstonine, ajmalicine, vindorosidine, and vincristine. Additionally, the flower has the anthocyanin pigment rosinidin. Vincristine, vinblastine, and indole alkaloids are two significant antitumor terpenoids that contribute to the plant’s medicinal significance [55]. A recent study described a novel method for producing silver nanoparticles (Ag NPs) using *Catharanthus roseus* (*C. roseus*) leaf extract and the solution combustion synthesis (SCS) method. Fifty grams of fresh *C. roseus* plant leaves were cleaned with deionized water and then cut into small pieces. Two hundred fifty milliliters of deionized water was used for extraction, and the resulting extract was filtered. A fixed range of plant extract volumes, ranging from 1 to 10 mL, was introduced to 0.1 g of AgNO_3_. The mixture boiled at 500 °C, and ignition happened in just 5 min. This pattern persisted within the temperature range of 500 ± 10 °C, eventually yielding Ag NPs. In the final phase, the product was gradually cooled to room temperature after 40 min of calcination at 500 ± 10 °C. Interestingly, the MDA-MB 231 breast cancer cell line’s viability significantly decreased in a dose-dependent manner when exposed to the synthesized Ag NPs. Moreover, this study reveals a unique aspect of Ag NPs: they inhibit the inflammatory enzyme secretory phospholipase A2 (sPLA2), which is known to be a breast cancer biomarker [94]. Other recent research showed that using the MTT assay, the proliferative activity of hepatocellular carcinoma (HepG2) and normal human liver (THLE3) cells treated with *C. roseus* AgNPs was quantified. After being cleaned, the leaves were dried at 40 °C in an oven. In a conical flask, the leaves were first ground and then mixed with 50 g of double-distilled water to 1 L. After being incubated at 40 °C in a water bath for the entire night, the mixture was centrifuged for 15 min at 2000 rpm. After being freeze-dried, the filtered supernatant was prepared for use in the production of *C. roseus*-AgNPs. A 10% *C. roseus* aqueous extract and 5 mmol/L silver nitrate (AgNO3) solution made up the *C. roseus*-AgNPs. After that, the mixture was collected and centrifuged at 10,000 rpm for 15 min. The pellet was gathered and freeze-dried, and the supernatant was disposed of. The results showed that genes linked to stress, including MT, HSP, and HMOX-1, were expressed in response to *C. roseus* AgNPs. The MAPK, TNF, and TGF pathways, which cause apoptosis and cell cycle arrest, may have activated the cellular signaling pathways. The uptake of *C. roseus*-AgNPs by both clathrin-dependent and clathrin-independent endocytosis was indicated by the alteration of ARF6, EHD2, FGFR3, RhoA, EEA1, VPS28, VPS25, and TSG101 [95]. Furthermore, recent research assessed the potential activity of incensole acetate (IA), a substance that was isolated and identified from *Catharanthus roseus* essential oil using column chromatography and GC/MS analysis. It also examined the potential anticancer impact of an IA-biosynthesized nanoemulsion against breast cancer. By using the MTT and crystal violet assay, the IA-mediated nanoformulation demonstrated cytotoxicity against breast cancer cell lines at an effective concentration. In a rat model of DMBA-induced increased interleukin serum indicators, nanoemulsion treatment led to a significant improvement. Furthermore, the potential effects of IA biosynthesized nanoemulsion on biochemical parameters, oxidative stress markers, proinflammatory cytokines, and tumor growth profiling in cancer-induced rats lend support to its anticancer properties [96], as shown in Figure 3. However, Vinblastine’s adverse effects include bone marrow suppression (which causes deep ulcers), gastrointestinal toxicity, significant vesicant (blister-forming) activity, and extravasation damage. Those with bacterial infections should not take this medication. Because vinblastine has been shown in animal studies to be embryotoxic, it should not be taken while pregnant. Breastfeeding is also not recommended because it may be secreted into breast milk. Vincristine’s main side effects are constipation, hyponatremia, peripheral neuropathy, and hair loss. The ingestion of *Catharanthus* alkaloids intrathecally carries a risk of death. There are instances of severe encephalopathy and demyelination of the spinal nerves leading to ascending paralysis, causing excruciating pain and finally death [97,98].

### 2.6. Curcuma longa

*Curcuma longa*, commonly referred to as “*Curcuma domestica*”, is a perennial herbaceous plant that belongs to the Zingiberaceae family, which also includes ginger [99]. It is extensively grown throughout tropical Asia’s south and southwest. The cuisines of Iran, Malesia, India, China, Polynesia, and Thailand all heavily incorporate turmeric, which is used as a spice and affects the texture, color, and flavor of food [100]. Despite having over 300 active components, the primary biologically active component of this plant is derived from its root and is either yellow or orange in color. This material is known as curcumin, which is the source of this plant’s therapeutic qualities [101,102]. Curcumin causes apoptosis in several tumor cell lines and in several animal models, including those of lung, liver, stomach, colon, breast, and esophageal cancer, among others; it inhibits tumor invasion and metastasis [103]. Additionally, curcumin triggers redox reactions in cells that produce reactive oxygen species (ROS), which causes the tumor cell membrane’s apoptosis receptors to become overexpressed. Additionally, curcumin can increase p53’s expression and activity, which reduces the growth of tumor cells and promotes apoptosis. Additionally, curcumin exhibits a strong inhibitory effect on NF-κB and COX-2 activity, which are implicated in the upregulation of antiapoptotic genes, like Bcl-2 [104]. The proinflammatory transcription factor NF-κB is essential for the growth of breast cancer cells. It controls the expression of proteins involved in cellular signaling pathways and over 500 distinct genes, which leads to the emergence of cancer and inflammation. Chemicals that can interact with NF-κB through its inhibition can be applied to cancer treatment. Curcumin demonstrated the capacity to inhibit the NF-κB-inducing genes, thereby influencing the proliferation and invasion of breast cancer cells [105,106]. The EGFR family tyrosine kinase receptor human epidermal growth factor receptor 2 (HER2) is another target that affects the growth of breast cancer cells. Given that the overexpression of HER2 contributes to the development of numerous cancer types, it is thought that HER2 may be a target for cancer therapy drugs. Curcumin can suppress breast cancer cell lines by blocking HER2-TK, either by itself or in conjunction with its analogs. Immuno-liposome encapsulation increased the selectivity of its HER2-suppressive action [107,108]. Curcumin can also obstruct the EGFR cell signaling pathway, which is a family of receptor tyrosine kinases linked to cancer cell migration, adhesion, proliferation, and differentiation. Through the reduction of EGFR signaling and the levels of EGFR and Akt, curcumin inhibited the growth and proliferation of breast cancer cells [109,110]. Curcumin has been shown to have the ability to alter the expression of miRNAs in breast cancer cells, which are non-coding sequences of 18–22 nucleotides implicated in several diseases, including cancer. The expression of tumor-suppressive (miR-15a, miR-16, miR-34a, miR-146b-5p, and miR-181b) and oncogenic (miR-19a and miR-19b) miRNAs in breast cancer cells was influenced by curcumin. Consequently, it was noted that apoptosis was induced and tumorigenesis and metastasis were suppressed [111]. Curcumin demonstrated its therapeutic efficacy in the treatment of lung cancer by downregulating NF-κB in human lung cancer cell lines A549 and by inhibiting JAK2 activity through its action on the JAK2/STAT3 signaling pathway. Moreover, curcumin upregulated microRNA-192-5p and suppressed the PI3K/Akt signaling pathway to reduce cell growth and cause apoptosis in human non-small cell lung cancer cells [112,113]. Curcumin demonstrated its therapeutic effect in colorectal cancer by interfering with multiple cell signaling pathways. Curcumin inhibited the growth of the in vitro cultured HT 29 cell line and rat colorectal carcinogenesis induced by DMH (1,2-Dimethylhydrazine) by suppressing the PPARγ signal transduction pathway. Furthermore, curcumin inhibited the expression of p53, pre-mRNA processing factor 4B (Prp4B), and cyclooxygenase-2 (COX-2) [114]. Previous research has suggested that curcumin inhibits the cell cycle and speeds up apoptosis to stop the spread of colorectal cancer by interfering with transcription factor E2F-1 and thymidylate synthase. This resulted in the downregulation of NF-κB and other survival pathways, which inhibited the cell cycle. Additionally, curcumin induced the G1 phase of the cell cycle by downregulating the kinase CDK2 [115]. Furthermore, curcumin inhibited cell proliferation rather than encouraging cell apoptosis in colon cancer cells SW480, thereby targeting the WNT/catechin pathway through a decrease in miR-130a expression. Additionally, curcumin inhibited colon cancer cell proliferation and induced apoptosis by targeting the miR-491/PEG10 pathway [116,117]. 

### 2.7. Glycyrrhiza glabra

**Figure 4:**
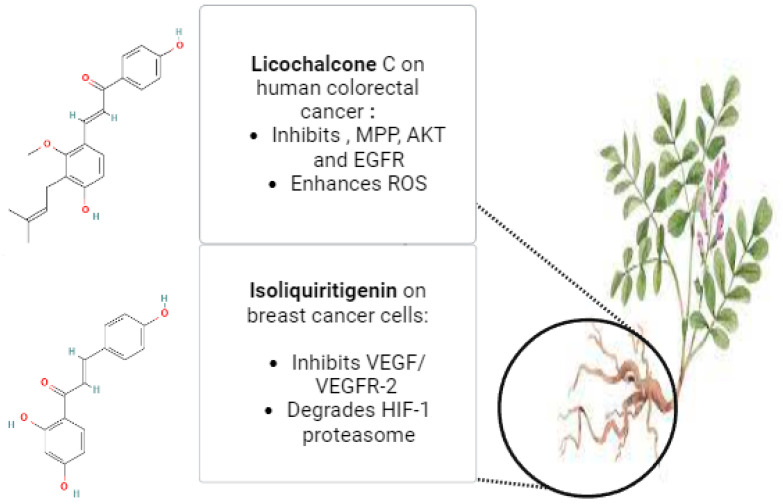
The potential anticancer effects of Licochalcone C and Isoliquiritigenin from *Glycyrrhiza glabra* roots (The molecular structures of the chemical compounds were retrieved from https://pubchem.ncbi.nlm.nih.gov/, accessed on 21 April 2024).

*Glycyrrhiza glabra*, the scientific name for licorice, is a member of the Leguminosae family. Herb *G. glabra* is widely used in Ayurvedic medicine. This herbaceous plant grows throughout Asia and parts of Europe [118]. Licorice contains a variety of substances, including proteins, amino acids, simple sugars, polysaccharides, mineral salts, pectin, starches, sterols, gums, and resins [119,120]. Approximately 300 flavonoid compounds and 400 other compounds have been isolated from licorice. Triterpenoid compounds are the primary active ingredients of glycyrrhizin, glycyrrhetinic acid, and their derivatives. Human metabolic processes can transform glycyrrhizin into glycyrrhetinic acid [121]. Isoliquiritigenin (ISL), which has demonstrated a direct inhibitory effect on malignancies, such as cervical, hepatoma, colon, breast, prostate, and other types of cancers, is one of the most active ingredients in glycyrrhiza roots. ISL can also prevent the development, propagation, and migration of multistage carcinogenesis processes by stimulating the cell cycle, apoptosis, autophagy, anti-angiogenesis, and other processes [29]. Through increased HIF-1 proteasome degradation and direct interaction with VEGFR-2 to decrease its kinase activity, ISL inhibited the expression of VEGF in breast cancer cells. ISL treatment for breast cancer has demonstrated its ability to inhibit both the growth of breast cancer and neo-angiogenesis. Furthermore, ISL inhibited VEGF/VEGFR-2 and raised the apoptosis ratio with negligible side effects. Consequently, it is plausible that ISL directly interacted with VEGFR2 to inhibit its kinase activity and increase HIF-1 proteasome degradation to decrease VEGF production in breast cancer cells [122]. Licochalcone C (LCC) is a chalcone compound that comes from the *Glycyrrhiza inflata* root. A previous investigation evaluated the antiproliferative properties of LCC in human colorectal carcinoma HCT116 cells, as well as HCT116-OxR (oxaliplatin-sensitive and -resistant HCT116 cells). HCT116 and HCT116-OxR cell growth was strongly and specifically inhibited by LCC. LCC was found to inhibit the kinase activities of both AKT and EGFR in an in vitro kinase assay. Through its targeting of EGFR and AKT, induction of ROS generation, and disruption of MMP, LCC demonstrated anticancer activity against both Ox-sensitive and Ox-resistant CRC cells. LCC could therefore be a useful therapeutic agent for the management of CRC cells that are resistant to oxygen [123]. Figure 4 illustrates the possible anticancer properties of isoliquiritigenin and licochalcone C from *Glycyrrhiza glabra* roots. The licorice root’s bioactive ingredient, 18β-glycyrrhetinic acid (18β-GA), possesses numerous antitumor characteristics. Using a PremoTM tandem autophagy sensor kit and Western blotting, the autophagy marker LC3-II conversion was studied. It was discovered that the 150-μM concentration of 18β-GA was inhibited. When rapamycin was added to the 18β-GA treatment, there was a significant increase in the phosphorylation level of jun-amino-terminal kinase (JNK). A JNK inhibitor (SP600125) prevented LC3-II accumulation, 18β-GA-mediated apoptosis, and the number of MCF-7 and T-47D colonies from forming. These findings suggest that 18β-GA in combination with 3-MA or rapamycin can either decrease or sensitize MCF-7 and T-47D cells to 18β-GA-induced apoptosis, respectively. By promoting apoptosis and activating JNK, 18β-GA modulated autophagy is cytotoxic to breast cancer cells of the luminal A subtype [124]. It has been demonstrated that licorice root’s natural compound, licochalcone A (LicA), possesses a range of anticancer properties. According to a recent study, LicA dramatically decreased the viability of endometrial cancer (EMC) cells and EMC-7 cells from EMC patients and caused apoptosis. The effects of LicA on human EMC cells were significantly attenuated by suppressing GRP78 expression. Based on the GRP78-mediated ER-stress pathway, LicA causes EMC cells to undergo apoptosis. This suggests that LicA may be used as an anticancer treatment for EMC [125].

### 2.8. Hibiscus

In the Malvaceae family, *Hibiscus* is a large genus of flowering plants. *Hibiscus rosa-sinensis* is a significant *Hibiscus* that is grown all over the world [126]. The primary chemical constituents of *Hibiscus sabdariffa* Linn. include tannins, carbohydrates, flavonoids, hibiscin, and steroids. Ascorbic acid, minerals, particularly calcium and iron, anthocyanins, and mallic acid are all present. Because *Hibiscus sabdariffa* Linn. contains a lot of phenolic compounds, it is a good antioxidant to treat several illnesses, including cancer, diabetes, liver, kidney, and neurological conditions [127]. It has been observed that dried leaf extract of *Hibiscus* containing saponins and phenols demonstrated proliferation inhibitory effects in prostate cancer cells and that flower extracts of the plant containing cyanidin-3-glucoside and anthocyanin significantly inhibited the growth of HeLa cervical cancer cells. Furthermore, in vitro and in vivo aberrant vascular smooth muscle cell migration and proliferation are inhibited by *Hibiscus sabdariffa* leaf polyphenols (HLP) [128]. Additionally, *Hibiscus* (HS) significantly affects nuclear factor-κβ (NF-κβ) and controls the expression of genes related to a multitude of processes, including angiogenesis, inflammation, migration, proliferation, and survival of cells. The majority of cancer types exhibit increased activity or constitutive activation of NF-κβ signaling, which is crucial for the advancement of carcinogenesis. Research has demonstrated that HS extract or its constituents inhibit NF-κβ activity by regulating the delicate equilibrium between the inhibitor of NF-κβ and its expression. For example, administration of HS leaf extract inhibited the ability of prostate cancer cell lines to bind NF-κβ DNA. (LN-Cap) [129]. It has been demonstrated that *Hibiscus* anthocyanin extract upregulates p53 in prostate and colon cancer cells. Anthocyanin can trigger the transcription of p21 and p27, which is a broad-spectrum CDK inhibitor that stops cancer cells from entering the cell cycle [130]. In a dosage-dependent manner, *Hibiscus* extract was able to specifically induce apoptosis in both triple-negative MDA-MB-231 and estrogen-receptor-positive MCF-7 breast cancer cells. When compared to treatment alone, the extract improved the induction of apoptosis by chemotherapeutic treatment (taxol and cisplatin) in MDA-MB-231 as well as ER+ breast cancer cells. Human promyelocytic leukemia cells (HL-60) were exposed to HS anthocyanin extract, which increased p38 phosphorylation and t-Bid, Fas, and FasL expression in a dose- and time-dependent manner [131]. A subset of tumor cells known as cancer stem cells (CSC) can both initiate and promote tumor recurrences. Tumor suppressors, like the p53 gene, are inactivated and oncogenes are overexpressed during the transformation process, which leads to cell proliferation and the transformation of cells into stem cells. The growth, angiogenesis, migration, invasion, and intracellular hypoxia-inducible factor-1 α (HIF1-α) of HCC, a type of CSC, were all inhibited by the phytochemical extracts of *Hibiscus*. Significant results demonstrated that *Hibiscus* phytochemicals, such as kaempferol and luteolin glycosides, flavonoids, polyphenols, caffeic acid, catechins, saponins, polysaccharides, triterpenoids, alkaloids, glycosides, and phenols (quercetin and luteolin), and can induce apoptosis in various cancers [132]. A recent study looked at the cytotoxic potential and mode of action of *Hibiscus sabdariffa* L. (HAs) extracts in colorectal cancer cells that contain anthocyanins in vitro. The findings demonstrated that extracts rich in *Hibiscus* anthocyanins induced apoptosis in human colorectal cancer cells by activating several AMPK signaling pathways. This resulted in the mitochondria’s releasing cytochrome C, which in turn caused the intestinal cancer cells to cleave caspase-3 and activate apoptosis [133].

### 2.9. Kalanchoe blossfeldiana

*Kalanchoe* species, perennial plants belonging to the family Crassulaceae, are found in tropical regions. There are about 150 species in this genus, the majority of which are found in Africa, Brazil, and India. Numerous additional biological and pharmacological activities are present in *Kalanchoe* plants including immunomodulatory, antibacterial, antiviral, antiproliferative, antihistaminic, and antidiabetic effects [134]. Previous research used UHPLC-QTOF-MS to examine the phytochemical composition of the *K. daigremontiana* water extract and to calculate the extract’s cytotoxic potential against SKOV-3 human ovarian cancer cells. The extract significantly halted the cell cycle in the S and G2/M phases of SKOV-3 cells, induced depolarization of the mitochondrial membrane, and demonstrated strong antiproliferative and cytotoxic activity. Furthermore, the extract raised the cancer cell line’s oxidative stress level. The extract may cause cell death through the tumor necrosis factor receptor (TNF receptor), according to the real-time PCR analysis [135]. HeLa cells from cervical cancer were used to test the cytotoxic potential of bersaldegenin-1,3,5-orthoacetate, a bufadienolide steroid found in plants in the *Kalanchoe* genus. By suppressing HeLa cell proliferation in the G2/M phase of the cell cycle and inducing cell death through double-stranded DNA damage and reactive oxygen species overproduction, the compound had a strong effect on the cells (IC50 = 0.55 μg/mL). Moreover, neither a rise in caspase-3/7/9 activity nor a fall in the potential of the cellular mitochondria was seen in the treated cells. The treated cells had overexpressed NF-Kappa-B inhibitor genes, according to gene expression analysis [136]. In CL1-5 highly metastatic human lung cancer cells, the effects of three bufadienolides—kalantuboside B, kalantuboside A, and bryotoxin C—isolated from *Kalanchoe tubiflora* were assessed and characterized. It has been shown that these bufadienolides induce autophagy, which in turn causes p-mTOR to be downregulated and LC3-II, ATG5, ATG7, and Beclin-1 to be upregulated. Our research demonstrated that in CL1-5 lung cancer cells, autophagy serves as an alternate mechanism of bufadienolide drug action. This suggests that bufadienolides may be a viable therapeutic approach for treating highly metastatic human lung cancer [137]. 

### 2.10. Moringa oleifera

A common plant in the tropic and subtropical zones of Africa, Asia, and South America is *M. oleifera*, a member of the Moringaceae family. Typically, the green pods, flowers, and leaves are eaten cooked or raw. It has been noted that the leaves are highly concentrated in vitamins, minerals, and essential amino acids. The majority of the bioactive substances have been extracted from the various plant parts. These include glycosides like niazirin, niazimicin, and niacin; phenolic acids, like quinic acid and chlorogenic acid, which have significant antioxidant potential; and isothiocyanates, like moringin, which have antitumor, antihypertensive, antibacterial, and hypoglycemic properties [138]. A previous study was successful in synthesizing iron oxide nanoparticles (Fe_2_O_3_ NPs) stabilized with *Moringa oleifera* (M.O.). Using a variety of tests, the study sought to investigate the cytotoxic, antiproliferative, and antimicrobial properties of Fe_2_O_3_ NPs. The nanoparticles had a consistent spherical shape and were confirmed to have formed M.O. Fe_2_O_3_ NPs by their Fe and O content. Various concentrations of M.O. Fe_2_O_3_ NPs were observed to exhibit both cytotoxic and antiproliferative potential on MDA-MB 231 human breast cancer cells. The cytotoxicity result showed an IC50 of 69.7 μg/mL [139]. Other work aimed to find possible CDK-2 inhibitors that could be used to treat hormonal receptor-positive breast cancers by utilizing *Moringa oleifera*. Molecular docking, MM-GBSA, and molecular dynamics simulations were the in-silico techniques used to screen *Moringa oleifera* compounds and determine their anticancer potential against CDK-2 protein targets. To confirm the anticancer potential of *Moringa oleifera* leaf extract, the MTT assay was carried out in vitro using MCF-7 cancer cell lines. At 200 µg/mL of fraction B (ethyl acetate), a significant antiproliferative effect was seen, and cell viability was decreased to 40% [140].

### 2.11. Nerium oleander

The shrub *Nerium oleander* L., belonging to the Apocynaceae family, thrives in subtropical areas such as the Arabian Peninsula, Southwest Asia, and the Mediterranean Basin. Within the genus *Nerium*, it is the sole species. It has a long history in ancient Europe and is used as an ornamental plant in parks and gardens. It has also been used in traditional medicine in the Near East and Southern Asia as an anti-inflammatory, antidiabetic, and anticancer herbal medication as well as a herbal remedy against indigestion, malaria, leprosy, and mental illnesses [141]. Because of its high phytochemical content, *N*. *oleander* shows a wide range of biological and pharmacological activities. Carbohydrates, flavonoids, alkaloids, steroids, cardiac glycosides, and tannins are abundant in *Nerium* leaves. Cinnamic acid makes up a large portion of the leaf’s polyphenol content, while epicatechin, catechin, and chlorogenic acid are present in smaller amounts. Apart from phenolic compounds, 2.3% crude polysaccharides are also present in the leaves; the major polysaccharides found are arabinose, galacturonic acid, galactose, and rhamnose. Phenol, tannin, flavonoid, coumarin, alkaloid, phlobatannin, and triterpene are abundant in *N. oleander* flowers. Numerous studies on *N. oleander*’s phytochemical composition and biological activity have been published in the literature. The antibacterial properties of *N. oleander* root bark tissue are caused by active ingredients like oleandrigenin and odoroside B [142,143]. In previous research, a hydroalcoholic extract from *Nerium oleander* leaves (containing 4.75 ± 0.32% of cardenolides) was prepared by using an ultrasound water bath apparatus, and fresh N. oleander leaves were extracted with ethanol: water (1:1) at 60 °C for one hour. A rotary vacuum evaporator was utilized to remove the ethanol, and the leftover water solution underwent lyophilization, yielding an extraction yield of 2.3%. Then, its cytotoxic activity in A549 lung cancer cells was evaluated and compared to MRC5 nonmalignant lung fibroblasts in this communication. The outcomes demonstrated that when compared to the nonmalignant cell line, the cytotoxicity of the *Nerium oleander* extract against the cancer cell line was significantly higher. The glycolysis inhibitor dichloroacetate, which is presently being developed for use in cancer therapy, and the *Nerium oleander* extract significantly reduced the production of lactate and consumption of glucose in A549 cells [144]. Moreover, another study looked into Breastin, a standardized cold water leaf extract from *Nerium oleander* L., for its potential to fight cancer. Five out of six carcinomas and fourteen cell lines from hematopoietic tumors showed growth inhibition due to breastin. Odoroseide H and neritaloside’s cellular responsiveness, surprisingly, did not correlate with any of the other classical drug resistance mechanisms found in 59 tumor cell lines of the National Cancer Institute (NCI, USA). These mechanisms included ATP-binding cassette transporters (ABCB1, ABCB5, ABCC1, ABCG2), oncogenes (EGFR, RAS), tumor suppressors (TP53, WT1), and others (GSTP1, HSP90, proliferation rate). These findings suggest that Breastin may elude drug resistance. Breastin frequently correlated with drugs that inhibited mitosis, according to comparative analyses conducted on 74 tumor cell lines from the Oncotest panel using 153 anticancer agents. Protein expression profiles can be identified to predict the sensitivity or resistance of tumor cells to breastin constituents. Proteome profiling of 3171 proteins in the NCI panel revealed protein subsets whose expression significantly correlated with cellular responsiveness to odoroside H and neritaloside. In vivo, breastin moderately inhibited the growth of breast cancer xenograft tumors. Breastin’s potential for drug combination regimens is demonstrated by the remarkable prevention of tumor relapse observed with the combination of paclitaxel and Breastin, which differs from what was seen with paclitaxel monotherapy [145]. However, the poisoning effects of the plant or its active alkaloids caused varying degrees of hemorrhage, myocardial degeneration, and necrosis. They also caused lesions, infiltration of inflammatory cells into the portal spaces with scattered hepatocyte necrosis in the liver, and infiltration of cells with hemorrhage and severe negative changes in the lung. Additionally, it caused a prolonged P-R interval, sinus bradycardia, and arrhythmia in electrocardiogram recordings. A recent case study described a child who mistakenly consumed a *Nerium oleander* leaf instead of a guava leaf. The young patient arrived at the hospital in a state of shock, with poor sensorium, hypotension, and vomiting. The child experienced bleeding symptoms, myocardial dysfunction, hyperkalemia, and acute kidney injury. This incident emphasizes how dangerous it is to consume *Nerium oleander* and how crucial it is to stay away from them near residential areas [146,147].

### 2.12. Silybum marianum *L.*

*Silybum marianum* L. refers to a tree plant that is derived from the Greek words silybon or silybos, which mean “twig” or “stem.” Since ancient times, *S. marianum* L. Geert. of the Asteraceae family has been recognized as an herbal remedy [148]. This plant, native to the Mediterranean, has also spread to America, Australia, and East Asia. Flavonolignans, silychristin, isomer silybin, and silydianin make up silymarin, a lipophilic extract derived from milk thistle. Milk thistle seeds contain small amounts of polyphenolic compounds, 20–35% fatty acids, and taxifolin. An isomer compound of special flavonoid complexes called flavonolignans, silymarin, makes up 1.5–3% of the weight of dried fruit. Similanin, isosilybin, silydianin, iso-silychristin, silybin, and isosilybin are all forms of silymarin. Flavonolignans, which are the fruit’s chemical composition, combine with other flavonoids (such as quercetin, taxifolin, kaempferol, dihydro-coniferyl, apigenin, naringin, eriodictyol, and chrysoberyl), 5,7-dihydroxy chromone, dihydro-coniferyl alcohol (60%) acid (30%, oleic acid, 9% palmitic acid), tocopherol, sterols (such as cholesterol, cholesterol, stigmasterol, and sitosterol), sugar (arabinose, rhamnose, and xylose), and protein [149]. Treatment with milk thistle can be employed, its achene is used to make coffee, and its flowers, leaves, and roots have all been consumed as vegetables in European cuisines. Hepatotoxins, like alcohol, tetrachloromethane, toluene, xylene, and carbon tetrachloride, can be combated by silymarin and silybin. Silybin stimulated ribosomal RNA and DNA polymerase reactions as well as liver cell regeneration. Silybin recognized membranes, increased glutathione in human and liver cell lines, increased glutathione peroxidase, and regulated the generation of free radicals [150]. To screen and identify potential EGFR antagonists from the Chinese medicine *Silybum marianum* (L.), a recent study used the SNAP-tag-epidermal growth factor receptor (SNAP-tag-EGFR) cell membrane chromatography (CMC) model, which is an effective tool for studying ligand–receptor interactions and screening active ingredients in traditional Chinese medicine. The positive control medication recognized was used to confirm the system’s applicability. They screened four possible EGFR antagonists from the Chinese herb *Silybum marianum* (L.). They were recognized as isosilybin, silydianin, silychristin, and silybin. Additionally, a CCK-8 assay was used to preliminary confirm their pharmacological activity. The growth of EGFR-overexpressing cells is inhibited by the four active ingredients, and they had good EGFR binding. The findings confirmed the possible antitumor effects of *S*. *marianum* (L.)on EGFR, and identified silydianin, silychristin, silybin, and isosilybin as main chemical components [151]. A previous study examined the anti-inflammatory and antioxidant properties of *Silybum marianum* L. undifferentiated callus extracts in both malignant and normal skin cells. A multistep extraction process was used to extract bioactive substances, yielding extracts with a wide range of active substances with various chemical and polar properties: 70%, 50% ethanol, and water. An ultrasonic water bath with three 10-min cycles was used for extraction, and the supernatant was filtered out afterward. The extracts were dried and resuspended to a concentration of 100 mg dry extract/mL in dimethyl sulfoxide (DMSO) before being used. A notable decrease in IFN-γ-induced IL-6 release was observed in HEKn and A431 cells after pre-treatment with *S. marianum* at concentrations varying from 15 to 125 μg/mL [152]. For many years, silymarin, a combination of flavonolignans extracted from the plant *Silybum marianum* L., was utilized in traditional herbal medicine. Biogenic methods, in particular the plant-based synthesis of selenium nanoparticles (SeNPs), are thought to be among the most effective ways to treat cancer because they are nontoxic, safe, and environmentally friendly. Si-SeNPs demonstrated a notably greater cytotoxic effect on gastric cancer cells (AGS) in comparison to silymarin while remaining non-toxic to normal cells. Si-SeNPs induced the expression of Bax/Bcl-2, cytochrome c, and caspase protein cleavage, which is linked to mitochondria-mediated apoptosis signaling in AGS cells, according to real-time PCR and Western blotting analysis. Furthermore, Si-SeNPs-inhibited PI3K/AKT/mTOR pathways were significantly linked to autophagy and apoptosis signaling in AGS cells, according to an agonist assay using a PI3K activator [153]. Furthermore, previous findings have shown the cytotoxic and morphological effects of silymarin on prostate cancer DU145 cells as well as its function in the CXCR1 and Slit2/Robo pathways. H&E stain was used for morphological assessment after 24-, 48-, and 72-h cytotoxicity tests were conducted to analyze the dosage of silymarin. Following that, analyses of the proteins SLIT2, ROBO1, and CXCR4 were performed using Western blot and immunocytochemistry. Based on MTT analysis, silymarin’s IC50 values against DU145 cells for 24-, 48-, and 72-h treatments were 315, 126, and 70 µM. Apoptotic body formations, membrane blebbing, and condensed, kidney-shaped, and eccentric nuclei were among the apoptotic hallmarks seen in H&E. In comparison to the control group, silymarin boosted the expressions of SLIT2 and ROBO1 and decreased CXCR4 [154]. Moreover, mothers of preterm infants were randomly assigned to receive either a commercial product (Piùlatte Plus, Milte) containing 5 g of a mixture of silymarin, phosphatidylserine, and galega (goat’s rue) as a placebo (5 g of lactose) or the combination once a day. The study was double-blinded. It is said that phosphatidylserine increases silymarin’s bioavailability. From day 3 to day 28 postpartum, either the drug or a placebo was administered. Mothers used a breast pump to express milk as needed at night and every two to three hours during the day. Measurements of milk production were made on postpartum days 7, 14, and 28. The treated group produced 200 mL of milk on average every day, while the control group produced only 115 mL [155]. However, 40 mothers who were nursing were surveyed in Australia and 40 of them were using milk thistle as a galactagogue. On a Likert scale, mothers generally assessed milk thistle as being in the range of “slightly effective” to “moderately effective.” Ten percent of moms who took milk thistle reported negative side effects, the most frequent of which were weight gain, nausea, and dry mouth [156].

### 2.13. Taraxacum officinale

The flowering perennial *Taraxacum officinale*, which is known as dandelion has long been used to treat cancer, diabetes, and hepatic, renal, and stomach disorders. Beneficial components can be found in its fruit (seed pods), roots, and leaves [157]. Although it originated in Eurasia, it has lately been introduced to other regions of the world [158]. Several beneficial bioactive phytochemicals, including lactones, sesquiterpenes, triterpenes, alkaloids, phenolic acids, and flavonoids (luteolin, isoquercitrin, and caffeic acid), are found in dandelion. Research has indicated that dandelion root has antitumor properties in breast, liver, pancreatic, lung, and colorectal cancers [159,160,161]. A previous study demonstrated the dose- and time-dependent inhibition of cell growth and proliferation by hydroalcoholic dandelion extract (HADE). HADE used autophagy and apoptosis to cause the death of 4T1 breast cancer cells. As the concentration of HADE rose, the quality of DNA fragmentation improved. As the concentration of HADE increased, the secretion of NO decreased. Autophagy and apoptosis in cancer cells induced by HADE were validated by gene expression analysis. Bax, Bax/Bcl-2 ratio, p53, Beclin-1, and Atg-7 overexpression as well as Bcl-2 downregulation were also evident in treated cancer cells [162]. A recent work assessed the effect of one of *Taraxacum*’s triterpene extracts, taraxerol (TRX), on the migration and invasion of MDA-MB-231 cells. The findings demonstrated that, in a time and concentration-dependent manner, TRX could block the migration and invasion of triple-negative breast cancer cell lines, MDA-MB-231 cells, with MAPK3 proving to be the most promising target and able to combine with TRX stably. Furthermore, TRX was found to block MDA-MB-231 cell migration and invasion by blocking the ERK/Slug axis. Furthermore, the suppressive effect of TRX on MDA-MB-231 cells was partially reversed by an ERK activator (tert-butylhydroquinone, TBHQ) [163].

### 2.14. Urtica dioica

*Urtica dioica* is an unusual herbaceous perennial flowering plant known as stinging nettle in the genus *Urtica* and family Urticaceae, is commonly found in Asia, Africa, and Europe, and has a long history of being used as food and traditional medicine [164]. Nettle leaf extract is one of the herbal remedies that worked well together in clinical, experimental, and trial settings. This plant is highly well-known, and its stems, leaves, and roots have been used for a very long time [165]. The variety of secondary metabolites found in *Urtica dioica* (*U. dioica*) may contribute to its benefits, and the geographic conditions, as well as taxonomic, morphological, and genetic factors, which seem to have a significant impact on the species’ content. Specifically, several investigations have examined and verified *Urtica dioica* as a beneficial source of phenylpropanoids and flavonoids [166]. The MCF-7 human breast cancer cell line was used to examine the activity of the aqueous extract of *U. dioica* leaves. The extract from *U. dioica* showed antioxidant and antiproliferative properties. Significant cell death was seen in a dose-dependent manner after 24, 48, and 72 h of exposure to varying concentrations of the *U. dioica* extract, with an IC50 value of 2 mg/mL concentration after 72 h of treatment [167]. Other investigation involved the application of *U. dioica* aqueous extract of leaves to two human breast cancer cell lines, MCF-7 (which has positive estrogen and progesterone receptors and wild-type P53) and MDA-MB-231 (which has negative estrogen and progesterone receptors and mutated P53). Following a 72-h exposure, the treatment’s cytotoxicity was verified, with IC50 values of roughly 2 mg/mL for both breast cancer cell lines. Particularly in MCF-7 cells, the *U. dioica* extract induced apoptosis and raised Bax expression levels. It is noteworthy that the gene expression of two other proteins, adenosine deaminase (ADA) and ornithine decarboxylase (ODC1), has been demonstrated to be impacted by *U. dioica* extract [168]. Investigations were conducted on *U. dioica*’s effects in a rat model of breast cancer induced by N-methyl-N-nitrosourea. Rats were fed an aqueous extract of *U. dioica* (50 g/kg powdered) for 5.5 months. Next, an assessment was conducted on the formation of mammary gland cancer, antioxidant enzyme activities, and lipid peroxidation. In rats with mammary tumors created by *U. dioica*, there was a decrease in lipid peroxidation and an increase in catalase antioxidant enzyme activity. In addition, the results showed a lower incidence of breast cancer formation along with a lower quantity of cancer masses [169]. Recent research showed that 3,4-divanillyltetrahydrofuran (DTH) was obtained by extracting the roots of *Urtica dioica* against prostate cancer cells. DTH’s cytotoxicity was also assessed using the WST assay on LNCaP and PC3 cells to determine the harmful effects of DTH on various androgen mechanisms. As demonstrated by the outcomes of cell assays, depending on androgen sensitivity, DTH lignan exhibits distinct behaviors, such as being more toxic to LNCaP cells than PC3 cells [170]. Several studies have assessed how different concentrations of methanolic extract of *Urtica dioica* (MEUD) affect the viability, pattern of death, and expression of the gene linked to apoptosis in normal human dermal fibroblast (HDF), hepatocarcinoma (HepG2), and colon cancer (HCT116) cell lines. With an IC50 value of approximately 410 and 420 μg/mL, MEUD demonstrated antiproliferative effects on HepG2 and HTC116 cells, respectively, after 48 h (*p* < 0.001). HepG2 and HTC116 cells showed apoptotic cells, but HDF did not. Furthermore, when HepG2 and HTC116 cells were treated with varying concentrations of MEUD, an increased level of the BAX/BCL-2 ratio was seen. By altering the expression of BAX and BCL2, MEUD may affect hepatocarcinoma and colon cancer cell lines at particular doses and alter the rate of their proliferation [171].

### 2.15. Withania somnifera

Historically, *Withania*, a member of the Solanaceae family, has been recognized for its ability to modulate the immune system. It also goes by the names “Indian ginseng” and “winter cherry”. *Withania somnifera* (L.) Dunal.’s dried roots are the source of Indian ginseng, also known as Radix Withaniae or winter cherry. This family of plants is called Solanaceae [172]. *Withania somnifera* L. is one of the twenty-three species in the genus *Withania*. In India, hilly parts of Punjab, Himachal Pradesh, and Jammu are among the northwest regions where it is grown at an elevation of 1500 m. Steroid lactones known as “withanolides,” such as withaferin A, 27-deoxy withaferin A, withanolides I–XI, and somniferous A–C, are included in *Withania*. Alkaloids, such as anaferine, anhydride, cuscohygrine, dl-isopelletierine, 3-tropyltigloate, and tropine, are also present. Additional substances that function as antistress agents include acyl steryl glucosides, sitoindosides VII–X, and withaferin A. Among *W*. *somnifera*’s aerial parts are 5-dehydroxy withanolide-R and withasomniferin-A. Iron is the primary microelement that ashwagandha is rich in. The main immune modulatory activities of therapeutic active compounds include sitosterol, daucosterol, withaferin-A, 2,3-dihydrowithaferin A-3β-O-sulphate, and withasomniferol-A [55,68]. A recent study investigated the remarkable efficacy of Withaferin-A (WA), a withanolide derived from *Withania somnifera* (Ashwagandha), in combating breast cancer. The main goal was to use network pharmacology predictions and in silico computational techniques to study the role of hedgehog (Hh) pathway proteins and WA’s intrinsic target proteins in breast cancer targeting. The final WA network and the protein–protein interaction network was created with the aid of the web tools Stitch and String. Cytoscape version 3.9 was then used to build the drug-target network of thirty common proteins. Computational analysis demonstrated the plausible anti-breast activity of WA, particularly about Hh proteins, suggesting stem-cell-level checkpoint inhibitions [173]. Recent research investigated the efficacy of phytochemicals from plants such as *Withania somnifera* that may have had anti-glioblastoma properties in this investigation. As a result, we virtually screened the substances against the EIF4A3 protein. Using Molecular Mechanics Poisson-Boltzmann Surface Area (MMPBSA), binding free energy analyses, and docking scores assessed with GOLD, AutoDock4.2, LeDock, and other tools, we further examined the efficacy of the compounds on our shortlist. Several *Withania*-specific compounds from *Withania somnifera* and one dietary compound, thiamine from *Castanea sativa*, have shown good ADMET properties, significant binding affinity towards the EIF4A3, and relatively good blood–brain barrier permeability among the phytochemical studies, providing a chance to create medication candidates that target glioblastoma brought on by overexpression of EIF4A3 [174]. A recent study examined the cytotoxicity of hydromethanolic extracts of *W. somnifera* fruits (W-F) and roots (W-R) and their chromatographic fractions against three normal oral mesenchymal cells [human gingival fibroblast (HGF), human periodontal ligament fibroblast (HPLF), and human pulp cells (HPC)] as well as oral squamous cell carcinoma (OSCC) cell lines [Ca9-22 (derived from gingiva), HSC-2, HSC-3, and HSC-4 (derived from tongue)]. Using a homogenizer, the roots and fruits of *W. somnifera* (each 200 g dry powder) were extracted separately using MeOH–H2O (8:2, *v*/*v*, 4 × 1.5 L). The extracts produced total extracts [W-R total (37.4 g, 19%) and W-F total (44.96 g, 22%, *w*/*w*)] after being vacuum dried at 40 °C using rotary evaporators. The Ca9-22 cell line was most sensitive to the root polar ethyl acetate (W-R EtOAc) and butanol (W-R BuOH) fractions. When comparing the untreated control group to the W-R EtOAc and W-R BuOH-treated oral cancer Ca9-22 cells, flow cytometric analysis demonstrated alterations in both morphology and the cell cycle profile. Apoptotic cell death was suggested by the morphological changes and subG1 accumulation induced by W-R EtOAc (125 μg/mL). Molecular modeling studies indicated that the most likely oncogenic targets of these molecules’ anticancer activity are BRD3 and CDK2. These results demonstrate the potential of *W. somnifera* as a cheap source of therapeutic agents for various oral cancers [175].

## 3. Conclusions

The word “cancer” describes a collection of illnesses that share the trait of aberrant cell growth and the capacity to invade or spread to other bodily regions. Around the world, these illnesses cause a sizable number of fatalities, and resistance to current cancer treatment protocols is the reason for their increasing ineffectiveness and myriad side effects. Finding a means to prevent cancer from developing in the first place and from spreading is therefore essential. In addition to developing new anticancer medications, new treatment approaches must be developed. Among these potential treatment approaches is the use of phytotherapeutics to target different molecular markers altered during cancer and, as a consequence, defend against cancer without or with minimal adverse effects. Thus, it is possible to deduce that phytotherapeutic compounds obstruct the different signaling pathways that initiate the development of distinct cancer types, opening the door for the creation and discovery of anticancer medications. In this review, 15 plants with cytotoxic and antitumor properties that have recently been linked to cancer treatment, their distribution, pharmacokinetics, mechanism of action, and possible side effects were presented and discussed. It is critical to develop new treatment approaches in addition to new anticancer drugs in which the secondary anticancer metabolites of these medicinal plants may be advantageous for chemotherapy protocols. This should be thoroughly investigated, combined with other medications, and clinically investigated to create novel anticancer medications.

## Figures and Tables

**Table 1:** The efficiency of various medicinal plants against different types of cancers (↑: increases, and ↓: decreases).

Medicinal Plant	Biological Outcome	Effective Pathway	Type of Cancer	Regional Source	Reference
*Annona muricata*	↓cell migration ↑apoptosis	↓Bcl-2 ↑P53 ↓EGFR	Tongue cancer Breast cancer	America Asia	[16,17]
*Arctium lappa*	↓cell migration ↑autophagy ↑apoptosis	↓S100A4 protein ↓PI3K ↓Bcl-2 ↑LC3B-II ↑Beclin-1	Cervical cancer Colon cancer Prostate cancer Breast cancer	Asia Europe	[18,19,20]
*Arum palaestinum*	↓proliferation ↑apoptosis	↓PI3K/AKT/mTOR	Liver cancer Prostate canceer	Asia Europe Africa Europe	[21]
*Cannabis sativa*	↑apoptosis ↓proliferation	↑caspase 3 ↓phosphoprotein kinase B	Colorectal cancer	Asia	[22]
*Catharanthus roseus*	↑apoptosis ↓proliferation	↑p53 ↑*M*Ts** ↑*HS* ↑*HMOX*	Lung cancer Breast cancer	Asia	[23]
*Curcuma longa*	↑apoptosis ↑autophagy ↓proliferation	↓YAP ↓SIRT1 ↑ROS ↑AMPK	Colorectal cancer Breast cancer Lung cancer	Asia	[24,25,26,27,28]
*Glycyrrhiza glabra*	↑apoptosis ↑autophagy	↑LC3-II ↓PI3K/RAC-α ↓Cyclin B1 ↓CDK1	Breast cancer, Prostate cancer Lung cancer Gastric cancer	Asia Europe	[29,30,31,32,33]
*Hibiscus*	↑apoptosis ↓proliferation	↑p53 ↓cyclin E/cdk2	Prostate cancer Cervical cancer Breast cancer	Asia America Europe Australia Africa	[34,35]
*Kalanchoe blossfeldiana*	↑apoptosis ↑autophagy	↑p53 ↓p-mTOR	Lung cancer Breast cancer Cervical cancer	Arica Asia America	[36]
*Moringa oleifera*	↑apoptosis ↑autophagy	↓NF-kB ↓Vimentin mRNA	Pancreatic cancer Breast cancer	Africa Asia America	[37,38,39]
*Nerium oleander*	↓proliferation ↑apoptosis	↓EGFR ↓TGF-β, VEGF, AFP, COX-2	Cervical cancer Lung cancer Liver cancer Colon cancer	Asia Europe	[40,41]
*Silybum* *marianum*	↑apoptosis ↑autophagy ↓proliferation	↑Bax/Bcl-2 ↓PI3K/AKT/mTOR ↓COX-2	skin cancer gastric cancer liver cancer	Asia America Australia	[42]
*Taraxacum officinale*	↑apoptosis ↓proliferation	↓MMP-9 ↓IL-1β ↑p53 ↑KAI1	Breast cancer	Europe America Asia Australia	[43]
*Urtica dioica*	↑apoptosis ↓proliferation	↑OCD1 ↓miR-21 ↓MMP1 ↓MMP9 ↓MMP13 ↓Bcl-2	Breast cancer Prostate cancer Gastrointestinal cancer	Asia North Africa North America Europe	[44,45,46,47]
*Withania* *somnifera* L.	↓proliferation ↑apoptosis	↑caspase 3 ↓RSK1, Akt1, and mTOR	Prostate cancer Breast cancer Ovarian	Asia	[48]

**Table 2:** The primary active components found in various medicinal plant parts.

Medicinal Plant	Main Active Component/s	Effective Plant Parts	Reference
*Annona muricata*	Acetogenins Alkaloids Phenolic compounds Flavonoids	Roots Bark Seeds Leaves	[49]
*Arctium lappa*	Lignans Caffeoylquinic acids Phenolic compounds Flavonoids Polysaccharides	Roots Seeds Leaves Fruits Stem	[50,51]
*Arum palaestinum*	Piperazirum Isoorientin Diketopiperazine Phenolic compounds Flavonoids	Flowers Leaves Roots	[52]
*Cannabis sativa*	Cannabidiol Tetrahydrocannabinol Cannabinol β-caryophyllene Cannabigerol	Fibers Oil Seeds	[53,54]
*Catharanthus roseus*	Vinblastine Vincristine Carbohydrates Alkaloids Flavonoids Saponins	Roots Flowers Basal stem	[55]
*Curcuma longa*	Curcumin	Rhizome	[56]
*Glycyrrhiza glabra*	Glycyrrhizin Glycyrrhetinic acid Isoliquiritigenin	Roots	[57]
*Hibiscus*	Anthocyanins Polysaccharides Flavonoids Organic acids	Flowers Leaves Seeds	[58]
*Kalanchoe blossfeldiana*	Flavonoids Anthocyanins Coumarins Phenolic acids Sterols	Roots Stem Leaves	[59,60]
*Moringa oleifera*	Flavonoids Tannins Alkaloids Glucosinolates Isothiocyanates Oleic acids	Leaves Flowers Seeds Pods Bark	[61,62]
*Nerium oleander*	Chlorogenic acid Rutin Quinic acid esters Oleandrin Flavonoids Carbohydrates Alkaloids Polysaccharides Tannins	Leaves Roots Bark Flowers	[63]
*Silybum* *marianum* L.	Silandrin Silybin silychristin Silydianin Silymarin Silymonin	Flowers Leaves Roots Achene	[64]
*Taraxacum officinale*	Sesquiterpene lactones Triterpene Chicoric acid Flavonoids Phenolic acids 4-hydroxyphenylacetate inositol esters	Roots Stem Leaves Flowers	[65,66]
*Urtica dioica*	Phenolic compounds Ferulic acid Lignans Phytosterols Isolectins Coumarins	Roots Stalk Leaves	[67]
*Withania* *somnifera*	Withamolides Sitoindosides Alkaloids	Roots Leaves Fruits	[68]

## Data Availability

This paper has all the data supporting the findings.
